# Exercise Enhanced Cardiac Function in Mice With Radiation-Induced Heart Disease *via* the FNDC5/Irisin-Dependent Mitochondrial Turnover Pathway

**DOI:** 10.3389/fphys.2021.739485

**Published:** 2021-11-11

**Authors:** Wuyang He, Yinghong Tang, Chunqiu Li, Xiaoyue Zhang, Shunping Huang, Benxu Tan, Zhenzhou Yang

**Affiliations:** ^1^Oncology Department, The Second Affiliated Hospital of Chongqing Medical University, Chongqing, China; ^2^Department of Geriatric Cardiology, The Second Affiliated Hospital of Chongqing Medical University, Chongqing, China

**Keywords:** FNDC5, irisin, radiation therapy, heart, aerobic exercise

## Abstract

**Background**: Despite the development of radiation therapy (RT) techniques, concern regarding the serious and irreversible heart injury induced by RT has grown due to the lack of early intervention measures. Although exercise can act as an effective and economic nonpharmacologic strategy to combat fatigue and improve quality of life for cancer survivors, limited data on its application in radiation-induced heart disease (RIHD) and the underlying molecular mechanism are available.

**Methods**: Fifteen young adult male mice were enrolled in this study and divided into 3 groups (including exercised RIHD group, sedentary RIHD group, and controls; *n* =5 samples/group). While the mice in the control group were kept in cages without irradiation, those in the exercised RIHD group underwent 3weeks of aerobic exercise on the treadmill after radiotherapy. At the end of the 3rd week following RT, FNDC5/irisin expression, cardiac function, aerobic fitness, cardiomyocyte apoptosis, mitochondrial function, and mitochondrial turnover in the myocardium were assessed to identify the protective role of exercise in RIHD and investigate the potential mechanism.

**Results**: While sedentary RIHD group had impaired cardiac function and aerobic fitness than controls, the exercised RIHD mice had improved cardiac function and aerobic fitness, elevated ATP production and the mitochondrial protein content, decreased mitochondrial length, and increased formation of mitophagosomes compared with sedentary RIHD mice. These changes were accompanied by the elevated expression of FNDC5/irisin, a fission marker (DRP1) and mitophagy markers (PINK1 and LC3B) in exercised RIHD group than that of sedentary RIHD group, but the expression of biogenesis (TFAM) and fusion (MFN2) markers was not significantly changed.

**Conclusion**: Exercise could enhance cardiac function and aerobic fitness in RIHD mice partly through an autocrine mechanism *via* FNDC5/irisin, in which autophagy was selectively activated, suggesting that FNDC5/irisin may act as an intervening target to prevent the development of RIHD.

## Background

As radiation therapy has been widely applied in thoracic tumor treatment, radiation-induced heart injury, which results in irreversible myocardial fibrosis and heart failure, has also attracted increased attention. The NRG oncology RTOG0617 trial reported that cardiac toxicity may impede the benefit of high-dose radiation therapy (RT) on overall survival (OS) in patients with non-small-cell lung cancer (Bradley et al., 2015). After long-term follow-up in the same cohort, the latest data further support the close relationship between heart V5 and OS, which suggests the potential damage of cardiac toxicity on the outcome after RT (Bradley et al., 2020). A linear correlation between the mean dose to the whole heart and cardiovascular risk was also observed in patients with breast cancer who received RT, without an apparent risk threshold remaining (McGale et al., 2011). Despite investigating RT techniques that minimize radiation exposure to the heart, early identification of and interventions for these complications remain to be reported.

Recent views have extended the cardiovascular toxicity induced by tumor treatment to the cardiovascular-skeletal muscle axis characterized by impaired cardiorespiratory fitness (CRF). The role of exercise as an effective nonpharmacological approach in controlling cardiovascular risk factors, halting the progression of cardiovascular disease and improving outcomes, has been widely accepted and limited, but growing data have supported the benefits of aerobic exercise in improving CRF and reducing cardiovascular risk in cancer survivors to relieve the “whole-body” damage caused by tumor treatment (Bhattacharya and Asaithamby, 2016), implying the potential of exercise in intervening radiation-induced heart disease (RIHD).

Irisin is an exercise-induced myokine that is cleaved from the transmembrane protein fibronectin type III (FNIII) domain-containing protein 5 (FNDC5) expressed in myocytes and may link the cardiovascular-skeletal muscle axis (Ma et al., 2021). Despite the role of FNDC5 as the precursor of irisin, FNDC5 could also act as the receptor of irisin based on its fibronectin type III domain containing the Arg-Gly-Asp (RGD) sequence (Main et al., 1992; Teufel et al., 2002; Erickson, 2013), which might mediate the autocrine or endocrine action of irisin. As cardiac damage induced by ionizing radiation often converges on mitochondria through DNA damage which is susceptible to ionizing radiation (Seol et al., 2012), the positive feedback of prolonged mitochondrial impairment and excessive ROS production may account for the long-term adverse effects following RT (Yahyapour et al., 2017; Schofield and Schafer, 2021). Irisin has recently been reported to be involved in the differentiation of mouse embryonic stem cells by promoting mitochondrial integrity (Nazem et al., 2017). Additionally, irisin could act as an oxidative stress scavenger and alleviate doxorubicin-induced cardiotoxicity (Liu et al., 2019; Zhang et al., 2019). However, the regulatory role of exercise-activated FNDC5/irisin in halting the accumulated RT-induced mitochondrial damage to cardiomyocytes remains unknown. Therefore, we aimed to investigate the protective role of aerobic exercise in RIHD and its potential regulatory role in mitochondrial function *via* the FNDC5/irisin pathway.

## Materials and Methods

### Animals

Fifteen young adult male C57BL/6 mice (6weeks old, weighing 12–15 g) were purchased from the animal laboratory of Chongqing Medical University. The posterior evaluation of sample size was conducted by one-way analysis of variance F test using PASS 15.0.5. The total sample size of 6 mice (*n* =3 groups) has achieved 100% power to detect the difference of means of irisin levels [K(Means Multiplier)=1, Effect size *f* =7.68, 7.46, 4.51, α err prob.=0.05, Means=69.32, 25.67, 53.68, Standard Deviation=2.35, 2.42, 4.00] among three groups. As 15 mice were enrolled in our study, the 100% power has been achieved to detect the difference among the means using an F test with a 0.05 significance level. To adjust the effect of confounders, such as feeding conditions and environmental factors, five mice were included per group, considering that five mice in a group were kept in the same cage with a controlled room temperature of 22±2°C under a 12-h light/dark cycle and given free access to water and food. Since estrogen is a protective factor against the risk of cardiovascular disease and heart disease, which has rarely occurred in female populations in earlier life stages (Iorga et al., 2017), we excluded female mice from the current study. In order to ensure the replicability, five samples/group was set to evaluate the effect of exercise on body weight, grip strength, cardiac function, aerobic fitness, and mitochondrial function and three samples/group with three duplicates/sample was set to evaluate the effect of exercise on the expressions of FNDC5/irisin and mitochondrial turnover markers.

All experimental procedures were conducted according to the ARRIVE guidelines and were approved by the Animal Care and Use Committee of Chongqing Medical University No. 2021053.

The mice were randomly divided into three groups: the control group (*n*=5), sedentary RIHD group (*n*=5), and exercised RIHD group (*n*=5). While the mice in the control group were kept in cages without irradiation, those in the exercised RIHD group underwent aerobic exercise intervention. All mice were sacrificed on the 21st day after exposure of the heart to X-ray irradiation. The mice in the sedentary RIHD group remained free within the cage and were sacrificed 21days after irradiation.

### Establishment of the RIHD Model

A single session of radiation exposure was conducted in this study, which has been previously reported (Kruse et al., 2003). The precordial area of each mouse was exposed to X-ray irradiation individually to establish a murine RIHD model. After all mice had been anesthetized with isoflurane anesthesia (2%) using a mask, the hair on the chest was removed, and irradiation was applied with a 6 MV X-ray beam energy at a dose of 20Gy/1 Fx with a 100-cm source surface distance in a 1×1-cm radiation field of the precordial area.

### Exercise Protocol

The moderate aerobic exercise protocol used with the exercised mice was performed on an animal treadmill (Zhongshi, Inc.), which has been described by our previous study (Wuyang et al., 2020). The angle of inclination of the treadmill is 0°. The period of the exercise trial is 3weeks. In the first week, an adaptation protocol was conducted (5days/week, 6m/min for 30min per day) to improve the reliance of the mice in following the protocol. During the remaining 2weeks, a moderate exercise protocol with moderate intensity was conducted [5days/week, 75% VO2 max (10m/min)] for 1h per day after the warm-up exercise (4m/min for 2min; Fernando et al., 1993). Once the mice were exhausted and could not reach the belt speed, they were allowed to rest for 30–60min, after which the protocols continued. The period of time to exhaustion was recorded at the end of each week.

### Echocardiography

Echocardiography (ESAOTE S.p.A., SL3116, Italy) was used to measure the cardiac function of all the subjects on the 21st day before euthanasia as previously described. Afterward, each mouse was anesthetized using isoflurane, and the hair on the chest was removed using a depilatory cream. The lower left ventricular end-diastolic dimension (LVEDD) and systolic left ventricular dimension (SLVD) were recorded *via* short-axis M-mode. The ejection fraction (EF) was calculated using the equation EF=stroke volume (SV)/end-diastolic volume (VD)×100%.

### Assessment of Grip Strength

An electronic grip strength meter (cat. 47200, Ugo Basil) was used to assess the grip strength of the forelimbs of all the mice at the end of each week. With mice fixed on a fence, the maximal grip strength was measured by slowly pulling at the base of the mouse tails. The procedure was repeated three times, and the highest value was recorded.

### Histological Analysis

After euthanasia, the blood was drained, and the tissue of the left ventricle without the septum was dissected and washed with ice-cold saline solution. The cardiac tissues were embedded in paraffin after fixation in paraformaldehyde fixative (4% paraformaldehyde) and then sliced into 5-μm-thick samples for further analysis. The method was performed according to a previously described protocol (Zeng, 2016). Hematoxylin and eosin (HE) and Masson staining were conducted according to a previously described protocol. The sections were analyzed by two independent single-blinded investigators under bright field microscopy (Thermo Scientific) and quantified using Image-Pro Plus 6.0.

### Assessment of Apoptosis

Cardiomyocyte apoptosis was assessed using a Cell Death Detection kit (4AF488 TUNEL assay, cat. No. FXP142-050, 4A Biotech, Inc.). The mean number of TUNEL-positive cells was assessed by analyzing 5 randomly chosen regions with Image-Pro Plus 6.0.

### Transmission Electron Microscopy

A 1-mm^3^ myocardium tissue block was fixed, dehydrated, and dyed with uranyl acetate and lead citrate as described previously (Cullen et al., 2010). Two experienced single-blinded investigators observed the samples by transmission electron microscopy (Hitachi7700, Japan), and ten random fields of each section were analyzed.

### Mitochondrial Function

#### Preparation of Mitochondria From Skeletal Muscle

Fresh myocardium tissues were homogenized with a glass homogenizer on ice within 1h after euthanasia. Isolation buffer (c3606, Beyotime, Shanghai, China) included in the tissue mitochondria isolation kit was used to isolate mitochondria from cardiac tissue. According to the manufacturer’s instructions for the kit, isolation buffer was added to homogenized tissue after the cardiac tissue was dissected and washed with ice-cold PBS solution and trypsin solution. Then, the supernatant was collected after the homogenate was centrifuged (600×g/min for 5min, 4°C) in a low-temperature centrifuge. After the supernatant was further centrifuged (11,000×g/min for 10min, 4°C), the mitochondria (sediment) was isolated. The mitochondrial pellet was resuspended in mitochondrial isolation buffer (Beyotime, Shanghai, China) as previously described (Ouyang et al., 2016).

#### Mitochondrial Protein Content

The mitochondrial protein content was measured with an enhanced BCA protein assay (Beyotime, China, P0010S) according to a standardized protocol.

#### Mitochondrial Membrane Potential (∆Ψm) Measurement

The mitochondrial pellet was stained with the JC-1 probe using a ∆Ψm assay kit with JC-1 (Beyotime, China, c2006). A modular multitechnology microplate reader (Thermo Scientific Varioskan LUX) was used to assess the absorption of monomers (fluorescence excitation was set at 490nm, and fluorescence excitation was set at 530nm) and J-aggregates (fluorescence excitation was set at 525nm, and fluorescence excitation was set at 590nm) according to the manufacturer’s protocol. Differences in ∆Ψm among groups were reflected by the ratio of J-aggregates/monomer.

#### ATP Assessment

After homogenization of fresh myocardial tissue on ice, the supernatant was collected, and adenosine 5′-triphosphate (ATP) levels were measured with a modular multitechnology microplate reader (Thermo Scientific^™^ Varioskan^™^ LUX) according to the guidelines of an ATP assay kit (Beyotime, China, S0026). The ATP content was normalized to the ATP protein concentration, which was measured with an enhanced BCA Protein Assay kit (Beyotime, China, P0010S).

#### Real-Time PCR

The PCR primers specific for FNDC5, mitophagy markers (PINK1, PARKIN, and LC3B), mitochondrial fission markers (DRP1 and FIS1), a mitochondrial fusion marker (MFN2), and a mitochondrial biogenesis marker (TFAM) are listed in Table 1. RNA was extracted from myocardial tissue using TRIzol reagent (TaKaRa, Inc.). The PrimeScript RT Reagent Kit (TaKaRa, Inc.) was used to conduct RNA denaturation and reverse transcription to generate cDNA. Target genes were assessed by quantitative real-time PCR with three duplicates for each sample. The 2-ΔΔCT method was used to analyze the data.

**Table 1:** PCR primer sequences.

Gene name	Sequence: 5'-3'(forward)	Sequence: 3'-5'(reverse)
GAPDH	CCTCGTCCCGTAGACAAAATG	TGAGGTCAATGAAGGGGTCGT
FNDC5	CACCTCAAGGCCAACTCTGC	CATGGTCACCTCATCTTTGTTCTT
PINK1	TGACCCACTGGACACTCGATG	TGGAGGAACCTGCCGAGAT
PARKIN	CCAGCAGTTAAACCCACCTACAA	AATTAAGACATCGTCCCAGCAAG
MFN2	AGATTACGGAGGAAGTGGAAAGG	GCATAGATACAGGAAGAAGGGGC
Drp1	ATTCCATTATCCTCGCCGTCAC	GTTCTGCGCCCATCTGGATC
LC3B	CGTCCTGGACAAGACCAAGTTC	GCAAGCGCCGTCTGATTATC
TFAM	GGCACCGTATTGCGTGAGAC	GGAAAAACACTTCGGAATACAGAC

*GAPDH, glyceraldehyde-3-phosphate dehydrogenase; FNDC5, fibronectin type III domain containing 5; PINK1, PTEN induced putative kinase 1; MFN2, Mitofusin-2; Drp1, Dynamin-1-like protein; TFAM, Transcription factor A mitochondria.*

#### ELISA

The irisin concentration in serum collected before euthanasia was measured *via* ELISA (MB-5653B, MB Biology, China) according to the manufacturer’s guidelines.

#### Statistical Analyses

The normality of continuous variables was tested by the Kolmogorov–Smirnov test. ANOVA was used to compare normally distributed variables (including body weight, grip strength, grip strength/body weight, ejection fraction, transverse diameter of myocardial fiber, mitochondrial protein concentrations, concentrations of ATP, time to exhaustion, and maximal velocity) among groups. Skewed variables (including mRNA expression of FNDC5, PINK1, LC3B, and DRP1) were compared by the Mann–Whitney U test. The data are shown as the mean±standard deviation (SD) or median with interquartile ranges for quantitative values. Linear relationships between irisin levels and the expression of mitochondrial turnover markers were assessed by Spearman correlation analysis. All data were analyzed using IBM SPSS Statistics version 25.0 and GraphPad Prism 6, and a value of *p* <0.05 was used to indicate significance. The one-way analysis of variance F test was conducted by PASS 15.0.5 (NCSS, LLC, version2015) to evaluate the sample size and the power of the current study.

## Results

### The Effect of Exercise on Body Weight and Grip Strength

**Figure 1:**
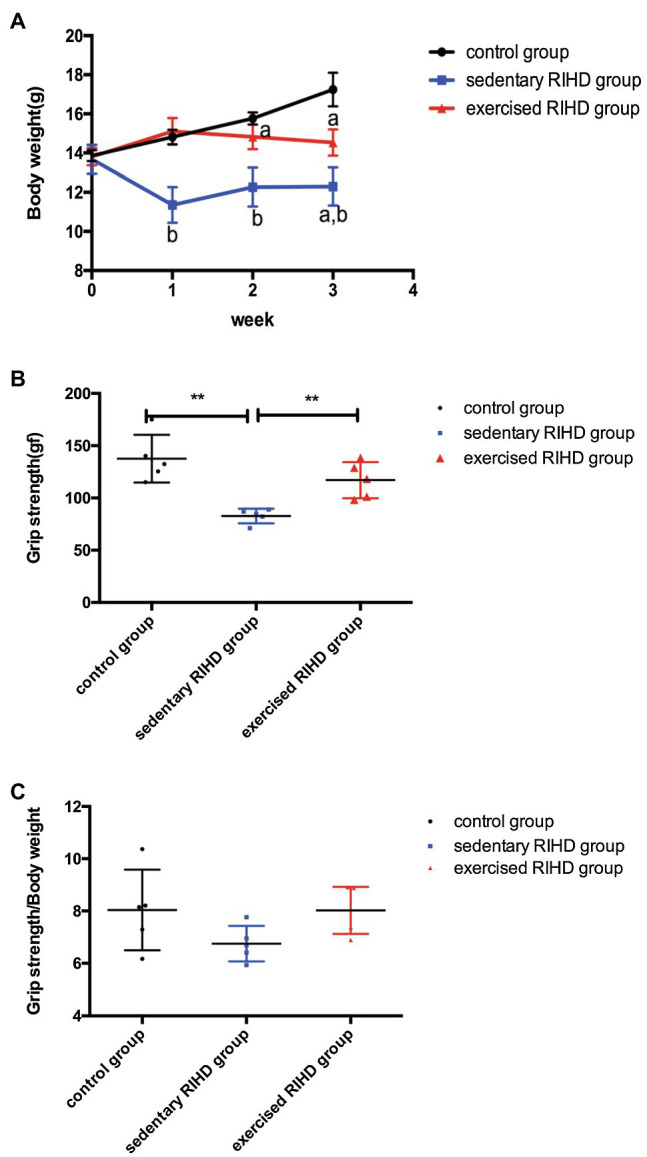
The effect of exercise on body weight and grip strength. **(A)** Body weights of the control group (black line, *n*=5), sedentary RIHD group (blue line, *n*=5), and exercised RIHD group (red line, *n*=5) at different time points. The results are presented as the mean and standard derivation. *p*^a^<0.05 compared with the control group, *p*^b^<0.05 compared with the sedentary RIHD group. Grip strength **(B)** and grip strength/body weight **(C)** at the end of the 3rd week after irradiation were compared by ANOVA. ^**^p<0.01.

While there was no significant difference in body weight among the groups at baseline, the body weight of the RIHD mice was significantly lower than that of the controls at the end of the 2nd and 3rd weeks after irradiation (2nd week: *F* value=33.22, degrees of freedom (between groups)=2, degrees of freedom (within groups)=12, 12.26±0.99 vs. 15.77±0.31g, 95%CI −4.48–−2.54, *p* <0.001; 3rd week: *F* value=40.84, degrees of freedom (between groups)=2, degrees of freedom (within groups)=12, 12.30±0.98 vs. 17.21±0.91g, 95%CI −6.10–−3.73, *p* <0.001), which indicates that RT may have affected murine body weight (Figure 1A). As the exercised RIHD group had higher body weight compared with the sedentary group at the end of each week (1st week:14.85(14.49–15.13) vs. 11.52(10.49–12.13) g, *p* =0.035; 2nd week: *F* value=33.22, degrees of freedom (between groups)=2, degrees of freedom (within groups)=12, 14.82±0.63 vs. 12.26±0.99g, 95%CI 1.59–3.53, *p* <0.001; 3rd week: *F* value=40.84, degrees of freedom (between groups)=2, degrees of freedom (within groups)=12, 14.54±0.67 vs. 12.30±0.97g, 95%CI 1.05–3.42, *p* =0.001; Figure 1A), exercise may impede the decrease in body weight induced by ionizing radiation.

As sedentary RIHD mice had significantly lower grip strength than controls at the end of the 3rd week (*F* value=13.21, degrees of freedom (between groups) =2, degrees of freedom (within groups) =12, 82.72±7.02 vs. 137.60±22.87 gf, 95%CI −57.78–−10.78, *p* =0.008), exercised RIHD mice had increased grip strength than sedentary RIHD mice (*F* value=13.21, degrees of freedom (between groups)=2, degrees of freedom (within groups)=12, 117.00±17.33 vs. 82.72±7.02 gf, 95%CI 10.78–57.78, *p* =0.008; Figure 1B). However, the difference in relative grip strength (grip strength/body weight) was not significant among the groups after adjustment for body weight (*F* value=2.25, degrees of freedom (between groups) =2, degrees of freedom (within groups)=12, *p* =0.149; Figure 1C).

### The Effect of Exercise on Cardiac Function and Exercise Fitness

**Figure 2:**
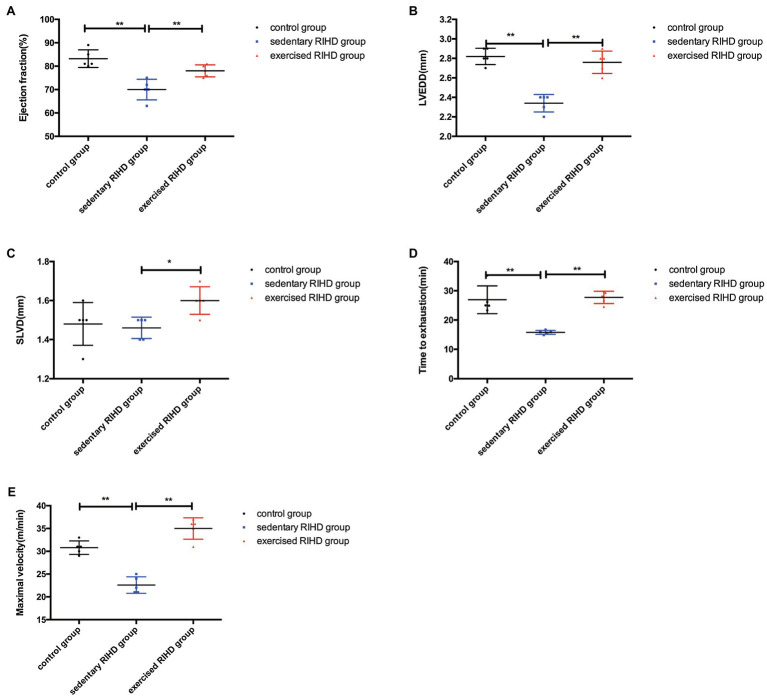
The effect of exercise on cardiac function and aerobic fitness in RIHD mice. Values for EF **(A)** and LVEDD **(B)** among groups were compared by ANOVA. **(C)** The Mann–Whitney U test was used to compare the SLVD among groups. **(D)** Time to exhaustion among the groups was compared by ANOVA. **(E)** Maximal velocity among the three groups was compared by ANOVA. *n*=5 samples/group. ^*^p<0.05, ^**^p<0.01. LVEDD, lower left ventricular end-diastolic dimension; SLVD, systolic left ventricular dimension; EF, ejection fraction.

The EF of mice in the sedentary RIHD group was significantly lower than that of controls (*F* value=15.70, degrees of freedom (between groups)=2, degrees of freedom (within groups)=12, 0.70±0.05 vs. 0.83±0.04, 95%CI −0.18–−0.08, *p* <0.001), but the exercised RIHD group had comparatively higher EF than the sedentary RIHD group (*F* value=15.70, degrees of freedom (between groups)=2, degrees of freedom (within groups)=12, 0.78±0.03 vs. 0.70±0.05, 95%CI 0.03–0.13, *p* =0.006; Figure 2A). While the sedentary RIHD group had significantly decreased LVEDD compared with the controls (*F* value=36.64, degrees of freedom (between groups)=2, degrees of freedom (within groups)=12, 2.34±0.09 vs. 2.82±0.08mm, 95%CI −0.61–−0.35, *p* <0.001), exercised RIHD mice had increased SLVD and LVEDD than that of sedentary RIHD mice (SLVD: 1.60(1.55–1.65) vs. 1.50(1.40–1.50) mm, *p* =0.035; LVEDD: *F* value=36.64, degrees of freedom (between groups)=2, degrees of freedom (within groups)=12, 2.76±0.11 vs. 2.34±0.09mm, 95%CI 0.29–0.55, *p* <0.001; Figures 2B,C). These data can support that exercise can improve the recovery of cardiac function following RT.

Considering that aerobic fitness, the time to exhaustion and maximal velocity were significantly decreased in the sedentary RIHD mice compared with the controls (time to exhaustion: *F* value=24.36, degrees of freedom (between groups)=2, degrees of freedom (within groups)=12, 15.79±0.67 vs. 26.94± 4.76min, 95%CI −15.33–−6.98, *p* <0.001; maximal velocity: F value=54.24, degrees of freedom (between groups)=2, degrees of freedom (within groups)=12, 22.60±1.82 vs. 30.80±1.48m/min, 95%CI −10.84–−5.56, *p* <0.001). Exercised RIHD group had increased time to exhaustion and maximal velocity than sedentary RIHD group (time to exhaustion: *F* value=24.36, degrees of freedom (between groups)=2, degrees of freedom (within groups)=12, 27.75±2.11 vs. 15.79± 0.67min, 95%CI 7.79–16.14, *p* <0.001; maximal velocity: *F* value=54.24, degrees of freedom (between groups)=2, degrees of freedom (within groups)=12, 35.00±2.35 vs. 22.60±1.82m/min, 95%CI 9.76–15.04, *p* <0.001), which suggests exercise may enhance the aerobic fitness of RIHD mice (Figures 2D,E).

### Exercise Could Improve Cardiac Myopathy Through the FNDC5/Irisin-Dependent Pathway

#### The Effect of Exercise on FNDC5/Irisin Expression

**Figure 3:**
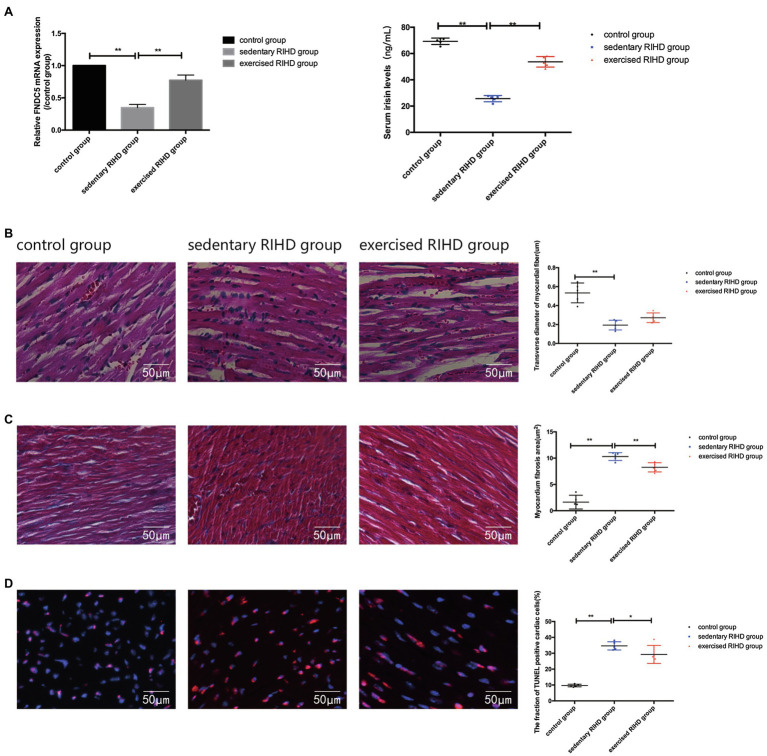
Effect of exercise on FNDC5/irisin expression and cardiac myopathy in RIHD mice. **(A)** Comparison of the relative mRNA expression levels of FNDC5/irisin among the three groups. **(B)** Hematoxylin–eosin staining of the three groups (*n*=3 samples/group). Magnification, ×400. The transverse diameters of the myocardial fibers between groups were compared by ANOVA. **(C)** Masson staining of the three groups (*n*=3 samples/group). Magnification, ×400. The quantified myocardial fibrosis areas among the groups were compared by ANOVA. **(D)** TUNEL-positive cells (indicated by the colocalization of red and blue fluorescence) were counted in three groups (*n*=3 samples/group). Magnification ×400. The fractions of TUNEL-positive cells were compared by ANOVA. ^*^p<0.05, ^**^p<0.01.

The mRNA expression of FNDC5 in the myocardium was decreased in sedentary RIHD mice compared with the controls (*F* value=180.68, degrees of freedom (between groups)=2, degrees of freedom (within groups)=12, 0.35±0.05 vs. 1.00±0, 95%CI −0.73 - −0.58, *p* <0.001), but exercised RIHD group had increased FNDC5 expression than sedentary RIHD group (F value=180.68, degrees of freedom (between groups)=2, degrees of freedom (within groups)=12, 0.77±0.08 vs. 0.35±0.05, 95%CI 0.35–0.50, *p* <0.001). Consistent with the alterations in FNDC5 expression, the serum irisin levels were decreased in the sedentary RIHD group compared with the normal controls (F value=8.323, degrees of freedom (between groups)=2, degrees of freedom (within groups)=11, 34.47±20.55 vs. 68.90±2.58ng/ml, 95%CI −53.17–−1.54, *p* =0.002) and exercised RIHD mice had significantly higher levels of irisin in contrast with sedentary RIHD mice (*F* value=8.323, degrees of freedom (between groups)=2, degrees of freedom (within groups)=11, 53.68±4.00 vs. 34.47±20.55ng/ml, 95%CI 1.54–36.88, *p* =0.036; Figure 3A).

#### The Effect of Exercise on Cardiac Myopathy

HE staining showed that sedentary RIHD group presented disarranged myocardial fibers, cardiomyocyte degeneration, nuclear condensation, eosinophilic enhancement, and more inflammatory cell infiltration than the controls. However, exercised RIHD mice had more ordered myocardial fibers with comparatively longer transverse diameters and decreased degeneration, cardiomyocyte nuclear condensation, and inflammatory cell infiltration compared to the sedentary RIHD group (Figure 3B).

Masson staining illustrated that the sedentary RIHD group exhibited a significantly higher myocardial fibrosis area than controls (*F* value=101.17, degrees of freedom (between groups)=2, degrees of freedom (within groups)=12, 10.30±0.74 vs. 1.63±1.31μm^2^, 95%CI 7.28–10.06, *p* <0.001), which was characterized by perivascular fibrosis. Exercise training decreased the myocardial fibrosis area in the RIHD mice (F value=101.17, degrees of freedom (between groups)=2, degrees of freedom (within groups)=12, 8.26±0.88 vs. 10.30±0.74μm^2^, 95%CI −3.44–−0.66, *p* =0.007; Figure 3C).

The fraction of TUNEL-positive cardiac cells was significantly higher in the sedentary RIHD group than the controls (*F* value=65.60, degrees of freedom (between groups)=2, degrees of freedom (within groups)=12, 34.64±2.57 vs. 9.68±0.84, 95%CI 19.97–29.96, *p* <0.001), revealing increased cardiomyocyte apoptosis in response to RT. The exercised RIHD group exhibited a significantly lower fraction of TUNEL-positive myocytes (*F* value=65.60, degrees of freedom (between groups)=2, degrees of freedom (within groups)=12, 29.27±5.67 vs. 34.64±2.57, 95%CI −10.37–−0.38, *p* =0.037), indicating that exercise training may decrease cardiomyocyte apoptosis (Figure 3D).

#### The Effect of Exercise on Mitochondrial Function and Mitochondrial Turnover

**Figure 4:**
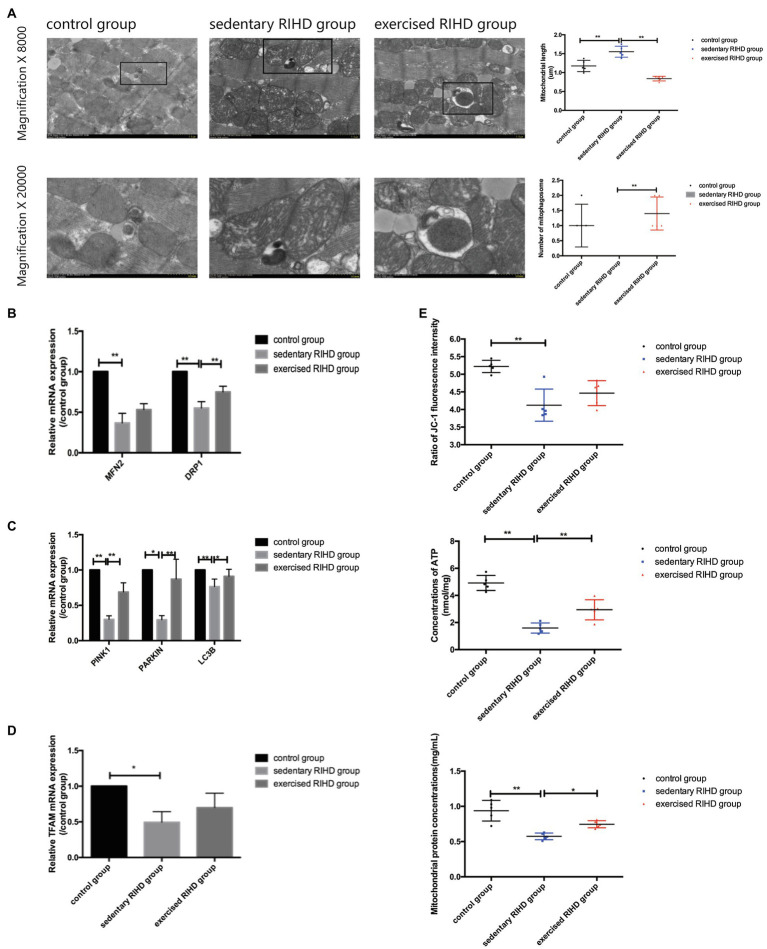
The effect of exercise on mitochondrial function and mitochondrial turnover markers in cardiomyocytes in RIHD mice. **(A)** The formation of mitophagosomes (indicated by the black square) was observed in the control and exercised RIHD groups. The mitochondrial length and number of mitophagosomes were quantified *via* Image-Pro Plus 6.0 (*n*=3 samples/group). ANOVA was used to compare the mitochondrial length among groups, while the number of mitophagosomes was compared using the Mann–Whitney U test. **(B)** Comparison of the relative mRNA expression of mitochondrial dynamics among the groups. **(C)** Comparison of the relative mRNA expression of markers of mitophagy markers in the groups. **(D)** Comparison of the relative mRNA expression of a mitochondrial biogenesis marker among groups. **(E)** The effect of exercise on the mitochondrial function of cardiomyocytes after irradiation. ANOVA was used to compare the mitochondrial membrane potential (ΔΨm), ATP production, and mitochondrial protein concentrations among the groups (*n*=5 samples/group). ^*^p<0.05, ^**^p<0.01.

Although the sedentary RIHD group had a significantly lower ∆Ψm (as indicated by the ratio of JC-1 fluorescence intensity) than the controls (*F* value=13.07, degrees of freedom (between groups)=2, degrees of freedom (within groups)=12, 4.12±0.46 vs. 5.22±0.17, 95%CI −1.58–−0.62, *p* <0.001), there was no significant difference in ∆Ψm between the exercised RIHD and sedentary RIHD groups. Interestingly, while sedentary RIHD mice had comparatively lower mitochondrial protein concentrations and a lower ATP content than controls (mitochondrial protein concentrations: *F* value=65.60, degrees of freedom (between groups)=2, degrees of freedom (within groups)=12, 0.58±0.05 vs. 0.94±0.15mg/ml, 95%CI −0.49–−0.23, *p* <0.001; ATP content: *F* value=41.62, degrees of freedom (between groups)=2, degrees of freedom (within groups)=12, 1.60±0.38 vs. 4.92±0.56nmol/mg, 95%CI −4.12–−2.52, *p* <0.001), exercised RIHD mice had significantly increased production of mitochondrial proteins and ATP (mitochondrial protein concentrations: F value=65.60, degrees of freedom (between groups)=2, degrees of freedom (within groups)=12, 0.75±0.05 vs. 0.58±0.05mg/ml, 95%CI 0.04–0.30, *p* =0.01; ATP content: F value=41.62, degrees of freedom (between groups)=2, degrees of freedom (within groups)=12, 2.94±0.74 vs. 1.60±0.38nmol/mg, 95%CI 0.55–2.14, *p* =0.003; Figure 4E).

In agreement with the alterations in mitochondrial function, the sedentary RIHD mice showed more swollen mitochondria and fewer mitophagosomes in myocardium than controls. Quantitative analysis of the electron microscopy images showed that exercised RIHD mice had a shorter mitochondrial length (*F* value=40.40, degrees of freedom (between groups) =2, degrees of freedom (within groups) =12, 0.84±0.06 vs. 1.55±0.15 um, 95%CI −0.88–−0.54, *p* <0.001) and more mitophagosomes (*p* =0.009) than those in the sedentary RIHD mice (Figure 4A), which indicates the enhanced mitochondrial fission and mitophagy in response to exercise. As a result, exercise was found to decrease ultrastructural damage to mitochondria and regulate mitochondrial turnover in RIHD mice.

Although the expression of a mitochondrial biogenesis marker (TFAM1; *p* =0.03), a mitochondrial fusion marker (MFN2; *p* =0.002), a mitochondrial fission marker (DRP1; *F* value=67.99, degrees of freedom (between groups)=2, degrees of freedom (within groups)=12, *p* <0.001), and mitophagy markers (PINK1, PARKIN, LC3B) was significantly decreased in sedentary RIHD mice compared to controls (PINK1: F value=95.50, degrees of freedom (between groups)=2, degrees of freedom (within groups)=12, *p* <0.001; PARKIN: *p* =0.002; LC3B: F value=10.00, degrees of freedom (between groups)=2, degrees of freedom (within groups)=12, *p* =0.017; Figures 4B–D), the mRNA expression of DRP1, PINK1, and LC3B was significantly elevated in exercised RIHD mice than that of sedentary RIHD mice (DRP1: F value=67.99, degrees of freedom (between groups)=2, degrees of freedom (within groups)=12, *p* <0.001; PINK1: F value=95.50, degrees of freedom (between groups)=2, degrees of freedom (within groups)=12, *p* <0.001; LC3B: F value=10.00, degrees of freedom (between groups)=2, degrees of freedom (within groups)=12, *p* =0.02), which indicates that exercise may regulate mitochondrial turnover by enhancing mitochondrial fission and mitophagy.

## Discussion

The increased cardiovascular risk associated with cancer care is often multifaceted and can be attributed to both impairment induced by tumor therapy and indirect toxic effects in the whole patient. Despite the protective role of aerobic exercise against cardiovascular risk and morbidity caused by anticancer therapy, which has been revealed by a growing amount of data (Codella et al., 2015; Scott et al., 2018a,b), the efficacy and regulatory mechanism of aerobic exercise in RIHD remain unknown. Our study adds to the literature by illustrating that aerobic exercise could promote the recovery of cardiac function and aerobic fitness following RT in RIHD mice. The cardiovascular benefit of aerobic exercise may occur through the regulation of mitochondrial turnover mediated by the FNDC5/irisin pathway.

In our study, aerobic exercise training significantly elevated the body weights of RIHD mice following RT. Since skeletal muscle mass accounts for most of the body mass, we speculate that aerobic exercise maintains body weight mainly by preventing muscle atrophy, consistent with previous data (Guo et al., 2019). Grip strength is a marker reflecting fatigue and frailty status, and aerobic exercise produced increased grip strength at the end of the 3rd week after RT, although the difference was not significant after adjusting for the effect of body weight. These results indicate that exercise may decrease fatigue and frailty following RT partly by increasing skeletal muscle mass. Anticancer therapy produces toxic effects extending to the heart-skeletal muscle axis, leading to impaired CRF and worsening outcomes (Stenehjem et al., 2016; Sweegers et al., 2019). We also confirmed that aerobic exercise strengthened aerobic fitness based on the reduced time to exhaustion and faster maximum velocity in RIHD mice that underwent exercise training.

While the alteration in EF on the 21st day after RT did not cause heart failure, the longer LVEDD in the sedentary RIHD mice suggested impairment of the contractile reserve and LV stiffness at an earlier stage of RIHD. Aerobic exercise can protect cardiac function by elevating both the contractile reserve and systolic function of the left ventricle, which is characterized by improvements in LVEDD, SLVD, and EF. This phenomenon may be further supported by the presence of less damage to cardiomyocytes as assessed by histological characterization, fewer perivascular fibrosis area, and decreased cardiomyocyte apoptosis observed in this study. These changes could slow the progressive process of cardiac dysfunction and remodeling caused by irradiation.

Since the mitochondrial DNA of cardiomyocyte is susceptible to irradiation (Piquereau et al., 2013; Hargitai et al., 2020), the prolonged damage to mitochondrial DNA can cause mitochondrial dysfunction and further disturbed the metabolic homeostasis of cardiomyocyte. Considering that the dynamic balance between mitochondrial biogenesis, fusion, fission, and mitophagy ensures initial mitochondrial quality control (MQC; Ni et al., 2015), the accumulation of abnormal mitochondria observed in myocardium of the sedentary RIHD group may support the disturbed mitochondrial dynamics in response to RT (Martinet et al., 2002; Puente et al., 2014). The increased number of mitochondria with a short mitochondrial length and enhanced production of mitophagosomes in exercised RIHD mice accompanied by improved mitochondrial function further support the notion that exercise can activate mitochondrial fission and mitophagy to clear damaged organelles and promote the recovery of mitochondrial function. Consistently, the expression of a mitochondrial fission marker (DRP1) and mitophagy markers (PINK1 and LC3B) was significantly elevated in RIHD mice following aerobic exercise, but mitochondrial biogenesis and fusion marker expression remained non-significantly changed. Thus, we can speculate that exercise can promote the selective degradation of damaged mitochondria to restore metabolic homeostasis in cardiomyocytes.

In parallel with the enhanced fission and mitophagy, the elevated FNDC5/irisin expression induced by aerobic exercise can support the potential role of FNDC5/irisin in regulating mitochondrial turnover. While the molecular mechanism by which exercise regulated RIHD remains unknown, the positive correlation between serum irisin concentrations and the expression of mitochondrial fission and mitophagy markers may imply that irisin can regulate mitochondrial turnover through autocrine activity. Considering that FNDC5 is characterized with its fibronectin type III domain containing the Arg-Gly-Asp (RGD) sequence which accounts for the cell adhesive property and is similar to other RGD receptors (Main et al., 1992), FNDC5 could act as a transmembrane receptor despite its role as the precursor of irisin (Teufel et al., 2002; Erickson, 2013). Recent data also supported the role of FNDC5/irisin in alleviating oxidative stress caused by doxorubicin-induced cardiac toxicity in an AKT/mTOR-dependent pathway, with improved the sensitivity of the tumor response to chemotherapy (Liu et al., 2019; Zhang et al., 2019). Although we were not able to confirm the interaction between irisin and mitochondrial turnover markers at the protein level, our data suggest that FNDC5 and irisin are involved in the preservation of mitochondria by aerobic exercise, which may promote the elimination of accumulated dysfunctional mitochondria following RT.

As aerobic fitness reflects whole-body health, the beneficial effect of FNDC5/irisin on exercise fitness reported in the current study may be attributed to not only the recovery of cardiac function, but also the regulation of skeletal muscle metabolism through the autocrine function of irisin (Gomarasca et al., 2020). Therefore, FNDC5/irisin can act as an “exercise medicine” for cancer survivors undergoing thoracic RT who are not able to participate in an aerobic exercise rehabilitation program because of barriers experienced by cancer survivors, such as pain, fatigue, and environmental issues (Blaney et al., 2013; Romero et al., 2018).

There are several limitations of this study. First, we did not demonstrate the interaction between FNDC5/irisin and mitophagy markers at the protein level. However, the relationship of FNDC5/irisin and mitochondrial turnover revealed in this study might inspire further investigation regarding the new function of FNDC5/irisin in regulating MQC. Second, we were not able to assess the dynamic process of mitochondrial turnover by electron microscopy. Nevertheless, the shorter mitochondrial length accompanied by an increased number of mitophagosomes and alterations in mitochondrial turnover markers further support enhanced fission and mitophagy.

## Conclusion

In conclusion, aerobic exercise may enhance the recovery of cardiac function and aerobic fitness by promoting mitochondrial fission and mitophagy through an FNDC5/irisin-dependent pathway. We have proven the efficacy of aerobic exercise in strengthening cardiovascular health in RIHD and revealed the potential of targeting FNDC5/irisin as an early intervention to prevent the development of RIHD.

## Data Availability Statement

The data that support the findings of the study are available from the corresponding author upon reasonable request.

## Ethics Statement

The animal study was reviewed and approved by The Animal Care and Use Committee of Chongqing Medical University.

## Author Contributions

WH and YT contributed equally to the conceptualization, study design, experiment conduction (including the establishment of animal model, western blotting, RT-PCR, histological analysis, and the assessment of mitochondrial function), data analysis, and drafting of the article. CL was responsible for conducting the exercise training of animal data analysis. ZY contributed to the conceptualization, supervision, project administration, and critical revision of the article. XZ, SH, and BT contributed to the establishment of the animal model. All authors agreed on the final content of the article.

## Funding

This study was supported by the National Natural Science Foundation of China (Grant No. 82100253 and 82002448), Chongqing Education Committee science and technology research project (KJQN202100463) and Chongqing Natural Science Foundation (Grant No. cstc2021jcyj-msxmX0268).

## Conflict of Interest

The authors declare that the research was conducted in the absence of any commercial or financial relationships that could be construed as a potential conflict of interest.

## Publisher’s Note

All claims expressed in this article are solely those of the authors and do not necessarily represent those of their affiliated organizations, or those of the publisher, the editors and the reviewers. Any product that may be evaluated in this article, or claim that may be made by its manufacturer, is not guaranteed or endorsed by the publisher.
